# 996 Burn Injury of the Oropharynx & Larynx, Functional SLP Assessment and Treatment

**DOI:** 10.1093/jbcr/iraf019.527

**Published:** 2025-04-01

**Authors:** Yekaterina Shemyakin

**Affiliations:** University of California Davis Medical Center

## Abstract

**Introduction:**

The role of the speech language pathologist in burn rehabilitation is focused on facilitation of safe oral diet, improving communication and speech as well as minimize oral contratures. Research supports a multidisciplinary team approach to coordinate the planning of reconstructive procedures, facilitate patient recovery, and optimize functional and aesthetic outcomes. Experience of the multidisciplinary team approach within the Firefighters Burn Institute Regional Burn Center, at UD Davis Medical center located in Sacramento CA, USA as it relates to evidence based approach to patient care.

**Methods:**

A literature review of the role and benefits of the speech language pathologist within the multidisciplinary team. Specific assessment and treatment approaches targeting dysphagia risks and management, effects of inhalation injury and endotracheal intubation on swallowing. Speech language pathologist role with burn patients with a tracheostomy and benefits of speaking valves for communication and swallowing. Review of the effects on communication and voice post burn injury.

**Results:**

Traditionally, occupational and physical therapists have managed orofacial contractures in this population with methods such as pressure garments, silicone masks, and oral splints. Research and data on functional units have shown that occupational and physical therapists require significant time to address the whole body needs of the burn care patient, calling for increased involvement from all professionals especially in larger total body surface area burns. Facial scarring and orofacial contractures result in functional deficits such as microstomia and limited range of motion affecting facial expression, oral intake, and articulation. The face may be grafted early, but rehabilitation exercises are often left for last. However, a general trend toward earlier intervention is now occurring. Medical Speech language pathologist play a vital role in therapeutic interventions to the face to improve functional swallow and speech outcomes

**Conclusions:**

Present data highlight that the speech therapy services need to be actively involved in the early and ongoing management of patients with burn injury in order to proactively support dysphagia risk and enhance swallow safety and voice recovery. Through targeted evaluation and interventions, medical speech therapist play a critical role in helping burn patients reclaim essential function and lead fuller lives. By working together, we can ensure every patient has the best possible change for recovery.

**Applicability of Research to Practice:**

Highlighting the need for increased speech therapy consults across the medical settings for burn patients.

**Funding for the Study:**

N/A

